# Hydrocracking of Waste Plastic Pyrolysis Oil and Light Cycle Oil (PPO/LCO) Blends in a Trickle-Bed Reactor: Catalyst Assessment and Operating-Condition Screening

**DOI:** 10.1021/acs.energyfuels.6c00240

**Published:** 2026-03-30

**Authors:** Iratxe Crespo, Tomás Cordero-Lanzac, Ana Cimiano, Roberto Palos, Alazne Gutiérrez

**Affiliations:** † Department of Chemical Engineering, University of the Basque Country UPV/EHU, PO Box 644, 48080 Bilbao, Spain; ‡ IKERBASQUE, Basque Foundation for Science, 48009 Bilbao, Spain

## Abstract

Plastic waste management is a major challenge for our society. The use of refinery facilities offers the possibility of treating them neatly or blending them with refinery streams to obtain products suitable for adaptability to market requirements. In this sense, this work investigates the production of automotive fuels through the hydrocracking of a blend composed of plastic-derived pyrolysis oil (PPO) and light cycle oil (LCO). First, three catalysts (PtPd/HZSM-5, PtPd/HY, and NiW/HY) were tested to study the influence of their acid, metallic, and structural properties in the hydrocracking reactions. This was performed using a PPO/LCO blend (20:80 in mass) at 400 °C, 60 bar, H_2_:feed volumetric ratio, 2000:1, space-time, 0.23 g h g_feed_^–1^, and time on stream (TOS), 8 h. The PtPd/HY catalyst exhibited good capacity for fuel production; its medium acid strength, superior hydrogenation capacity, and microporous structure favored gasoline production, reaching a selectivity of 81 wt %. With this catalyst, the effects of pressure and the amount of PPO in the blend were also assessed. At high pressures (80 bar), overcracking reactions were more likely to occur, while the conversion of heavy fractions remained constant. A similar trend was observed when the PPO loading was increased up to 40 wt %, the formation of light compounds was promoted to the detriment of the conversion of heavy fractions and gasoline formation.

## Graphical abstract

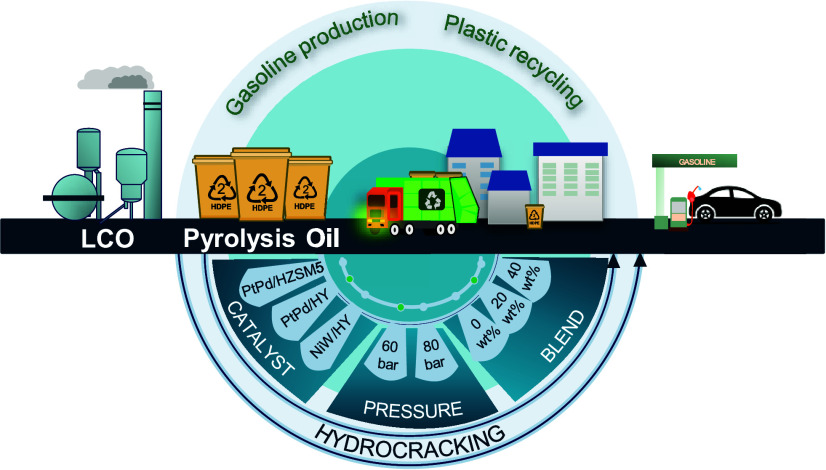

## 1 Introduction

Plastics are present in nearly every aspect of our lives. Their advantageous qualities —such as durability, strength, lightweight nature, cost-effectiveness, and versatility —have led to their widespread use in a variety of products, from everyday items to technical applications.^1^ As a result, global plastic production has steadily increased since the mid-20th century, reaching 413.8 Mt in 2023.^2^ However, the huge manufacturing combined with the typical short lifespan of plastic goods results in the massive disposal and accumulation of plastic waste. Moreover, the current waste management infrastructure is incapable of handling this overwhelming challenge, which has led to an alarming rate of plastic waste pollution.^3^ This does not only affect the environment, but it is also a serious problem for human health due to the transfer of toxic substances.^4^ Unless urgent mitigation measures are taken in this regard, the climate situation is expected to worsen in the coming years.

To mitigate plastic waste pollution and increase recycling rates, it is essential to develop effective recycling strategies and technologies that contribute to the circular economy of plastics. Among these technologies, thermochemical routes stand out as being particularly suitable. During gasification, plastics interact with a gasification agent, such as steam or oxygen, at high temperatures to produce high-value syngas.^5^ Nonetheless, this process has poor selectivity for products and requires significant capital investment and high energy consumption.^6^ On the other hand, pyrolysis, which is carried out by heat application in the absence of oxygen,^7^ breaks down large polymer chains into high-value chemicals such as monomers and hydrocarbons. Among the resulting products, the liquid known as plastic pyrolysis oil (PPO) attracts the most attention as it can be converted both into fuels and chemicals.^8,9^ However, to use the PPO as a feedstock for a chemical process, it must be submitted to an upgrading process, such as hydrotreating^10^ or hydrocracking.^11^ Indeed, a recent study has shown that the combination of pyrolysis with a hydrotreating-hydrocracking strategy has the potential to meet refinery naphtha specifications,^12^ suggesting this technology may emerge as a dominant chemical recycling technology for waste plastic for the next decade.

In this context, the possible use of refinery hydrocracking units for the upgrading of the PPOs could be a turning point in the management and valorization of waste plastics.^13^ But, considering the huge capacity of an average industrial hydrocracking unit, which could easily treat 50,000 bpd, it would not be a realistic and scalable approach to directly treat the raw PPO within these units.^14,15^ Instead, it should be blended with any of the refinery side-streams commonly used as feedstock for hydrocracking units, such as light cycle oil (LCO) or vacuum gasoil (VGO).^11^

However, most published studies to date have focused on the hydrocracking of neat plastics.^16,17^ Investigations into the hydrocracking of PPOs are scarce,^18,19^ and even scarcer the studies examining PPOs blended with conventional refinery streams.^20^ Besides, the majority of these studies employed discontinuous or semicontinuous batch reactors.^21,22^ This further restricts the up-scaling of the process from laboratory-bench to industrial-scale production, as the industrial hydrotreaters and hydrocrackers are known to be continuous trickle-bed reactors.^23^

In addition to the reactor configuration, the effectiveness of hydrocracking reactions is largely dependent on the catalytic system used. In this context, bifunctional catalysts, which combine metal and acid sites, are crucial for boosting the conversion and selectivity toward higher-value fractions. The support, typically a zeolite, has a decisive influence on the activity and selectivity of the catalyst, mainly through its porous structure and acidity.^24^ The metallic function is responsible for the hydrogenation reaction. These are promoted by noble metal catalysts, such as platinum or palladium.^25^ Also, transition metal catalysts, containing nickel, molybdenum, or cobalt, are commonly used in hydrotreatment processes due to their high activity in hydrogenation, hydrodesulfurization, and aromatic saturation reactions.^26^

Herein, this study seeks to advance the field in two key areas. First, it investigates the hydrocracking of HDPE-derived PPO blended with LCO, an area where research is still limited, despite the growing interest in upgrading these streams to increase gasoline yields. A previous study^27^ already explored the possibility of coprocessing PPO with VGO, demonstrating the feasibility of using this alternative feed to produce middle distillates. Industrial hydrocrackers, however, can be operated toward either gasoline or middle-distillates maximization depending on market conditions and the specific configuration of the unit. Here, we focus on the gasoline-oriented operating mode, using the gasoline yield and quality as sensitive indicators of catalyst behavior when processing the PPO/LCO blend. Second, a continuous trickle-bed reactor was employed to more closely approximate industrial operation. Furthermore, catalysts with different physicochemical properties were screened to select the catalyst with the best performance. The influence of the reaction pressure and PPO/LCO blending ratio on the product distribution and composition was also evaluated. Overall, the results highlight the advantages of cofeeding PPO with refinery side-streams and point to viable routes for the industrial valorization of waste plastic-derived oils.

## 2 Experimental Section

### 2.1 Catalyst Preparation

Three catalysts were prepared to evaluate the effects of the metal and the support on the process performance. Two of them were noble metal-based catalysts (PtPd) supported on HZSM-5 and HY zeolites (PtPd/HZSM-5 and PtPd/HY catalysts, respectively), whereas the third one consisted of transition metals (NiW) supported on HY zeolite (NiW/HY catalyst). Prior to metal loading, both HY and HZSM-5 zeolites were calcined at 550 °C for 15 h to stabilize the crystalline structure and guarantee thermal stability during the hydrocracking experiments.

The noble metal-based catalysts were prepared incorporating Pt and Pd on the HZSM-5 (CBV3024E, Zeolyst International) and HY (CBV712, Zeolyst International) zeolites. Metals were added by wet impregnation at 80 °C. Pt­(NH_3_)_4_(NO_3_)_2_ (Alfa Aesar) and Pd­(NH_3_)_4_(NO_3_)_2_ (Stem Chemicals) solutions were used, and their concentrations were adjusted to get 0.5 wt % of each metal in the final materials. The impregnated samples were first dried in a rotary evaporator, then dried overnight at 110 °C, and finally air-calcined at 450 °C for 2 h (5 °C min^–1^).^28^

The NiW/HY catalyst was prepared by adding Ni and W to the HY zeolite through wet impregnation. The salts used as metal precursors —Ni­(NO_3_)_2_ 6H_2_O and (NH_4_)_6_H_2_W_12_O_40_ *x*H_2_O— were both of 99.99 wt % purity and purchased from Sigma-Aldrich. The followed procedure has been explained before in a previous publication.^27^ In short, the Ni (nominal content of 4.5 wt %) was first added to the support in a rotary evaporator at room temperature. Then the material was dried for 18 h in an oven at 110 °C, and afterward air-calcined at 450 °C (5 °C min^–1^) for 3 h. W (nominal content of 22.5 wt %) was added to the already Ni-impregnated catalyst in the rotary evaporator. It was oven-dried for 12 h at 100 °C and finally calcined at 450 °C for 2 h (5 °C min^–1^).

### 2.2 Catalyst Characterization

Since the hydrocracking performance of catalysts depends on their physicochemical properties, they were characterized using several analytical techniques. Metal content was determined by wavelength-dispersive X-ray fluorescence (WD-XRF) using a PANalytical AXIOS spectrometer equipped with a Rh anode and three detectors (gas flow, scintillation, and Xe-sealed). The results were compared to those of well-established standards for rocks and minerals. Metal dispersion in the noble metal catalysts was determined by CO pulse chemisorption in a Micromeritics AutoChem II 2920 system coupled to a Blazer Instruments mass spectrometer. Before the measurements, the samples were reduced in a flow of 10 vol % H_2_ in Ar (100 cm^3^ min^–1^) at 300 °C (5 °C min^–1^) for 1 h and then cooled and stabilized at 60 °C. Afterward, CO pulses of 0.25 mL were introduced until saturation was reached. Metal dispersion was calculated by assuming a CO-to-metal stoichiometry of 1:1 for both metals.

N_2_ adsorption–desorption was carried out in an ASAP 2010 apparatus. Before the analysis, the samples were degassed under vacuum (2.7 10^–6^ bar) and 150 °C for 8 h to eliminate moisture and impurities. The experiments were performed at −196 °C with N_2_ in the 0.01:1 relative pressure interval. From the isotherm results, the specific surface area (*S*_BET_) was determined according to the simplified Brunauer–Emmett–Teller (BET) equation, the micropore volume (*V*_micro_ and *V*_meso_) by the *t*-plot method, and the pore size distribution using the Barrett–Joyner–Halenda (BJH) method.

Temperature-programmed desorption (TPD) of adsorbed NH_3_ was performed in an SDT 2960 Simultaneous DTA-TGA thermobalance (TA Instruments) connected in line to a mass spectrometer (Balzers Quadstar 422, Pfeiffer). First, the sample was pretreated (100 cm^3^ min^–1^ of 10 vol % H_2_ in Ar) at 450 °C (10 °C min^–1^) for 3 h (in a similar thermal treatment as for the reactions). Then, it was cooled to 150 °C; the gas was changed to N_2_ (40 cm^3^ min^–1^), and the injection of NH_3_ started. Once the sample was saturated, the NH_3_ injection was stopped and the physisorbed fraction was removed. The final step was the TPD of the NH_3_, for which the sample was heated to 600 °C (5 °C min^–1^). The density of acid sites was obtained from the amount of NH_3_ chemisorbed from thermogravimetric measurements.

The nature of the acid sites was measured by pyridine adsorption using Fourier-transform infrared spectroscopy (FTIR) in a Nicolet 6700 apparatus (Thermo Fisher). A catalyst pellet was prepared by using a Specac manual hydraulic press and then placed inside a Specac HT/HP cell. First, the moisture and the impurities were removed by heating the sample to 550 °C (10 °C min^–1^) under vacuum (0.82 bar) for 30 min. Then, it was cooled to 150 °C before the pyridine adsorption started. Several cycles of pyridine injection and evacuation were carried out until the sample was fully saturated. From the results, three bands were differentiated:^29^ the band at 1455 cm^–1^ attributed to Lewis acid sites; the band at 1495 cm^–1^ assigned to synergistic adsorption of pyridine in Lewis and Brønsted acid sites; and the band at 1545 cm^–1^ related to Brønsted acid sites.

### 2.3 Feeds

Light cycle oil (LCO), supplied by the PETRONOR S.A. refinery (Muskiz, Spain), is a byproduct of the refinery’s FCC unit. Although its boiling range is similar to that of commercial diesel (Table 1), its high content of total aromatics (72.6 wt %) and elevated sulfur compounds (10,212 ppm) render it unsuitable for direct blending into the commercial diesel pool.

**Table 1:** Main Physicochemical Properties of the LCO and PPO

	LCO	PPO
density, g cm^–3^	0.887	0.796
Simulated Distillation		
IBP–FBP, °C	140–421	70–515
*T*_50_–*T*_95_, °C	254–377	305–481
gasoline, wt %	26.2	26.5
diesel, wt %	61.3	33.2
gasoil, wt %	12.5	40.3
Elemental Composition, wt %		
C	89.6	84.6
H	9.4	13.4
N	0.1	0.3
O		1.7
S	1.0	
Lumped Composition, wt %		
paraffins	22.6	41.3
olefins	3.2	38.3
naphthenes	1.5	6.0
aromatics	72.6	14.4
monoaromatics	23.6	6.6
diaromatics	42.1	5.2
polyaromatics	6.9	2.5

Plastic pyrolysis oil (PPO), supplied by GAIKER Technological Centre (Zamudio, Spain), was produced in the pyrolysis of postconsumer high-density polyethylene (HDPE).^30^ According to the results in Table 1, the PPO had a wider boiling range than the LCO, together with a higher content of heavy compounds (40.3 wt %). However, it had a lower content of aromatics (14.4 wt %), since its nature is mainly paraffinic (41.3 wt %) and olefinic (38.3 wt %).

Two different PPO/LCO blends were prepared to assess the hydrocracking of the cofeeds: B20 and B40. The composition of the former was 20 wt % PPO and 80 wt % LCO, while in the latter, the content of PPO was 40 wt % and that of LCO was 60 wt %. Moreover, control reactions feeding pure LCO (B0) were carried out for the sake of comparison. The main physicochemical properties of the blends are available in Table S1 in the Supporting Information.

### 2.4 Reaction Equipment and Product Analysis

The PPO/LCO blends were pumped using a Gilson 307 HPLC piston pump into a fixed-bed Microactivity Reference (PID Eng&Tech) reactor operating under a trickle-bed regime, where hydrocracking reactions took place. For an in-depth description of the experimental setup and reaction procedure, please consult the published work.^31^ Before initiating the hydrocracking reactions, all catalysts were sieved to a particle size within the range of 0.125–0.3 mm and subsequently loaded into the reactor according to the methodology described by van Herk et al.^32^ The catalyst underwent *in situ* activation, wherein the noble metal-based catalysts were reduced at 400 °C for 4 h, using a gas mixture of 30 cm^3^ min^–1^ of H_2_ and 50 cm^3^ min^–1^ of N_2_. Conversely, the NiW/HY catalyst was subjected to sulfidation at 400 °C for 4 h under a 30 cm^3^ min^–1^ flow of H_2_S:H_2_ mixture (10 vol %).

The experiments were conducted at a temperature of 400 °C and pressures of 60 and 80 bar with a hydrogen-to-feed volumetric ratio of 2000:1. The space-time was set at 0.23 g h g_feed_^–1^, and the time on stream (TOS) was 8 h. The reaction products were collected in a Peltier cell at 0 °C, where the liquids condensed. Every hour, samples of both liquid and gas were taken for analysis.

Gas products were analyzed by gas chromatography in an Agilent Technologies 6890 GC equipped with an HP-PONA capillary column (50 m × 0.2 mm × 0.50 μm) and a Flame Ionization Detector (FID). For proper separation of the lightest compounds, the analysis started at cryogenic temperatures (−30 °C).

The different fractions in the liquid products were identified through simulated distillation analysis as per the ASTM D2887 Standard. This analysis was conducted using an Agilent Technologies 6890 GC gas chromatograph, which was equipped with a DB–2887 semicapillary column (10 m × 0.53 mm × 3 μm) and an FID detector. The liquids were classified into three fractions: gasoline, diesel, and gasoil, based on their boiling point ranges: 35–216 °C for gasoline, 216–343 °C for diesel, and above 343 °C for gasoil.

The composition of the liquid fraction was determined by using a comprehensive two-dimensional Agilent 7890 gas chromatograph (GC × GC) connected to a mass spectrometer (Agilent 5975 C inert XL MSD). The gas chromatograph was equipped with two columns linked by a flow modulator: a nonpolar DB-5 ms column (30 m × 0.25 mm × 0.25 μm) and a polar HP-INNOWax column (5 m × 0.25 mm × 0.15 μm), along with an FID detector.

The amount of coke deposited on the catalysts was determined through temperature-programmed oxidation (TPO) in a TA Instruments TGA-Q 500 thermobalance. The thermogravimetric analyses were performed under a 50 cm^3^ min^–1^ flow of an oxidizing atmosphere by heating the sample to 550 °C (5 °C min^–1^). This temperature was maintained for 30 min to ensure the total combustion of all of the carbonaceous deposits.

The catalyst’s performance was evaluated according to the conversion (*X*) of the heavy fractions present in the feed, the selectivity attained by each fraction (*S_i_*), and hydrodearomatization conversion (*X*_HDA_). These indices were defined as follows

1 $$X=\frac{{\left(m_{d\mathrm{iesel}}+m_{g\mathrm{asoil}}\right)}_{\mathrm{feed}}-{\left(m_{d\mathrm{iesel}}+m_{g\mathrm{asoil}}\right)}_{\mathrm{products}}}{{\left(m_{d\mathrm{iesel}}+m_{g\mathrm{asoil}}\right)}_{\mathrm{feed}}}100$$

2 $$S_{i}=\frac{m_{i}}{\sum_{i}m_{i}}$$

where *m_i_* is the mass flow of each *i* lump.

3 $$X_{\mathrm{HDA}}=\frac{{a\mathrm{romatics}}_{\mathrm{feed}}-{a\mathrm{romatics}}_{\mathrm{products}}}{{a\mathrm{romatics}}_{\mathrm{feed}}}100$$

## 3 Results and Discussion

### 3.1 Catalyst Properties

The textural properties of each catalyst were analyzed to understand their impact on the hydrocracking of the PPO/LCO blends. Figure 1a illustrates the N_2_ adsorption-desorption isotherms. The HY zeolite-based catalysts show isotherms that are a combination of types I and IV isotherms, characteristic of microporous catalysts, as they exhibit significant N_2_ adsorption at low relative pressures (*P*/*P*_0_ < 0.1).^33,34^ The hysteresis loops for HY-supported catalysts are classified as H4 type, typical of zeolite aggregates with narrow pores and substantial adsorption in the micropore range.^35^ On the other hand, the PtPd/HZSM-5 catalyst did not show a hysteresis loop, mainly adsorbs N_2_ in the microporous range, and shows some adsorption at very high relative pressure. This can be attributed to the presence of large mesopores.

**Figure 1:**
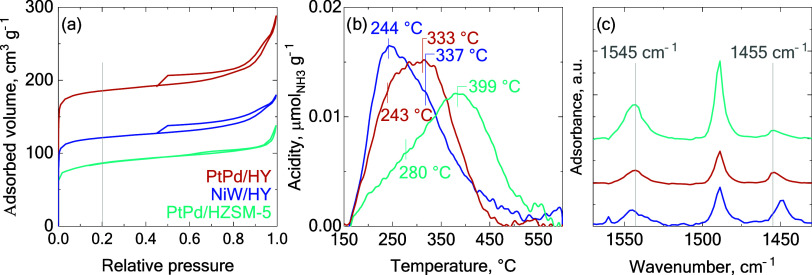
(a) N_2_ adsorption-desorption isotherms, (b) NH_3_-TPD profiles, and (c) FTIR spectra of adsorbed pyridine for the PtPd/HZSM-5, PtPd/HY, and NiW/HY catalysts.

Table 2 summarizes the results obtained from the N_2_ adsorption-desorption isotherms. The PtPd/HY catalyst exhibited the largest specific surface area (722 m^2^ g^–1^) and micropore volume (0.18 cm^3^ g^–1^). In contrast, the NiW/HY catalyst showed a significant decrease in both the specific surface area and the pore volume compared to the PtPd/HY catalyst despite the fact that the same commercial HY zeolite was used as the support for both catalysts. This result is a consequence of the high metal content of the NiW/HY catalyst. The PtPd/HZSM-5 catalyst had the lowest specific surface area and micropore volume, measuring 396 m^2^ g^–1^ and 0.15 cm^3^ g^–1^, respectively. These lower values for the PtPd/HZSM-5 catalyst can be attributed to the textural properties of the parent HZSM-5 zeolite used.

**Table 2:** Physicochemical Properties of the Zeolites and Catalysts

	HZSM-5	HY	PtPd/HZSM-5	PtPd/HY	NiW/HY
Metal Content^a^					
Pt, wt %			0.29	0.22	
Pd, wt %			0.53	0.33	
Ni, wt %					4.9
W, wt %					24.6
metal dispersion, %			16.7	22.6	
Textural Properties^b^					
*S*_BET_, m^2^ g^–1^	501	844	396	722	488
*S*_micro_, m^2^ g^–1^	452	774	353	668	427
*V*_micro_, cm^3^ g^–1^	0.16	0.31	0.15	0.26	0.18
*V*_meso_, cm^3^ g^–1^	0.05	0.16	0.09	0.18	0.09
pore diameter, nm	6.5	7.6	7.7	12.4	9.1
density of acid sites^c^, μmol_NH3_ g^–1^	610	639	530	576	561
B/L ratio^d^	8.6	4.3	7.3	3.7	1.0

a Measured by WD-XRF.

b Measured by N_2_ adsorption-desorption.

c Measured by NH_3_-TPD.

d Measured by Pyridine adsorption.

The characterization of the acid sites of the catalysts revealed comparable acid site densities; however, some differences in their acid properties became evident through the NH_3_-TPD profiles depicted in Figure 1b. The area under the desorption curves serves as an indicator of the acid sites density, while the peak maximum desorption temperature reflects the strength of the acid sites. A stronger chemisorption of the NH_3_ molecule on the acid sites of the catalyst requires a higher desorption temperature. According to established classifications,^36^ acid site strength can be classified as a function of the maximum desorption temperature: weak acid sites (zone I) appear between 150 and 250 °C, medium acid sites (zone II) desorb in the 250–350 °C range, and strong acid sites (zone III) manifest at temperatures above 350 °C.

Analyzing the shape of the NH_3_-TPD profiles, all of them can be deconvoluted into two Gaussian-type contributions, the maximum of which has been indicated in Figure 1b. The PtPd/HY and NiW/HY catalysts exhibited the following desorption peaks: the first located within zone I (243 and 244 °C, respectively), indicative of weak acid strength, and the second one in zone II (333 and 337 °C, respectively), denoting medium acid strength. In contrast, the PtPd/HZSM-5 catalyst displayed a minor peak at 280 °C and a significant peak at 399 °C, suggesting a considerable acid strength in spite of its lower density of acid sites (530 μmol_NH3_ g^–1^). Comparing the catalysts supported on the HY zeolite, the PtPd/HY catalyst demonstrated greater acid strength than the NiW/HY catalyst, which is attributed to a higher proportion of weak sites in the latter. This difference may be related to the higher metal content of NiW/HY, which overlaps the strong acid sites. Nonetheless, the total acid density was not much lower than that of PtPd/HY catalyst, so this difference could be also related to the interaction of the NiO and WO_3_ metal phases with the zeolite framework that might have formed additional Lewis acid sites, which increase the contribution of weak and medium acid sites.^37,38^

To obtain a comprehensive understanding of the acid properties, the Brønsted and Lewis acid sites were characterized by using FTIR spectroscopy of adsorbed pyridine. The spectra are illustrated in Figure 1c, while Table 2 presents the Brønsted to Lewis (B/L) acid sites ratio values. The observed B/L ratios align closely with the trends in acid strength, revealing that the PtPd/HZSM-5 exhibits a significantly higher ratio of 7.3, in contrast to the PtPd/HY catalyst with a ratio of 3.7, and the NiW/HY catalyst, which shows a markedly lower ratio (1.0). This might be related to the aforementioned formation of additional Lewis acid sites occurring during Ni and W salt impregnation.

Comparing these values with the results obtained for the raw zeolites (available in Table 2), a noticeable decrease in the B/L ratio is evident upon metal incorporation. This result could be linked to the preferential deposition of metal particles on Brønsted acid sites, as previously reported in the literature.^39^ This phenomenon is particularly pronounced when the two HY zeolite-supported catalysts are used. The greater metal loading in the NiW/HY catalyst strongly influences the B/L ratio, suggesting a direct correlation between the amount of incorporated metal and the loss and/or transformation of Brønsted acid sites.

The dispersion of Pt and Pd was determined by CO pulse chemisorption. The results indicate a higher metal dispersion for the PtPd/HY catalyst (22.6). This may be attributed to the slightly lower metal loading content, but most likely to the higher micropore volume of the HY parent zeolite (Table 2). It should be noted that dispersion was not measured for the NiW/HY catalyst because CO pulse chemisorption is not suitable for assessing the dispersion of the W species. Moreover, given the substantial differences in the metal content and active phase (PtPd metal and NiW sulfides), the values would not be directly comparable.

### 3.2 Product Selectivity and Composition

#### 3.2.1 Catalysts Screening

In a series of conducted experiments aimed at evaluating catalyst performance in the hydrocracking of the B20 blend, significantly different trends in the conversion and product selectivity are observed when using the three catalysts (Figures 2 and 3, respectively). Initially, the formation of gas fraction was predominant. However, as the reaction advanced, a partial deactivation of the catalysts was observed, with the deactivation rate and the steady-state conversion values being different for each studied catalyst (Figure 2). The decrease in the conversion led to a decrease in the gas selectivity and a subsequent increase in gasoline and diesel fractions (Figure 3). This trend was particularly marked for the PtPd/HZSM-5 and PtPd/HY catalysts, in contrast to that of the NiW/HY catalyst. The different behavior of the latter could be related to its weaker acid sites after the incorporation of a higher metal loading, as the conversion level stands between both noble metals-based catalysts along all of the TOS values (Figure 2). The catalyst deactivation observed in the early stages of the reaction is attributed to the deposition of coke, which hindered product conversion (Section 3.3). Apart from coking, the deactivation at early stages has been reported to be associated with metal poisoning caused by the sulfur heteroatoms of the feedstock.^40^ As the reaction progresses, a pseudo-steady-state is reached, where the catalyst deactivation becomes dominated by coke deposition, which outweighs this reported contribution of active-site poisoning by adsorbed species.^41^ This pseudo-steady-state is the consequence of reaching a pseudoequilibrium between the formation of coke species and the hydrocracking reactions of coke precursors.^42^

**Figure 2:**
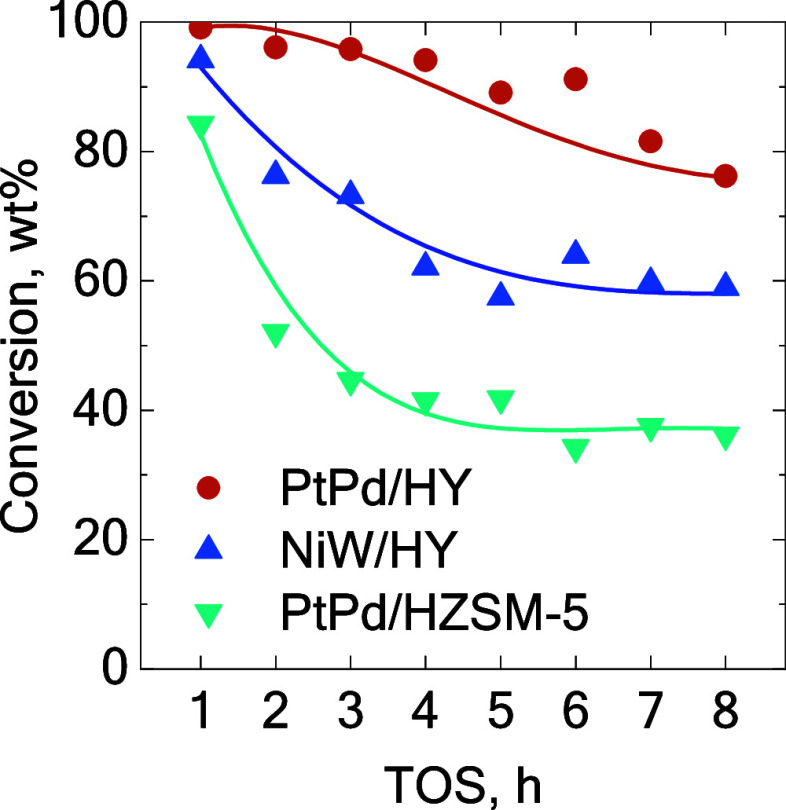
Evolution of the conversion with the TOS. Comparison between catalysts using the B20 blend at 400 °C and 60 bar.

**Figure 3:**
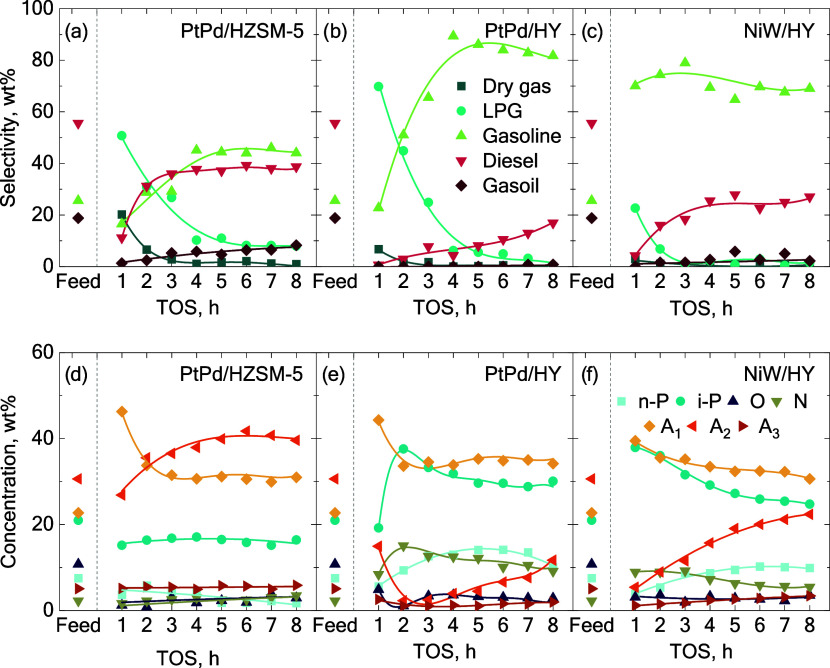
Evolution with the TOS of the products selectivity (a–c) and the PIONA composition (d–f) obtained in the hydrocracking of the B20 blend at 400 °C and 60 bar with PtPd/HZSM-5, PtPd/HY, and NiW/HY catalysts, respectively. Key: n-P, n-paraffins; i-P, i-paraffins; O, olefins; N, naphthenes; A_1_, monoaromatics; A_2_, diaromatics; A_3_, polyaromatics.

At the reaction onset, the PtPd/HZSM-5 catalyst achieved a total gas selectivity (dry gas and LPG) of 71.0 wt% (Figure 3a), while PtPd/HY surpassed 76.0 wt% (Figure 3b). Although the total gas selectivity was marginally higher with the PtPd/HY catalyst, the PtPd/HZSM-5 catalyst produced a higher concentration of dry gas (20 wt%). It should be noted that in hydrocracking, gaseous product formation can result either from thermal or cracking reactions via a β-scission mechanism.^43^ The higher gas selectivity of the PtPd/HZSM-5 catalyst can be ascribed to the inherent properties of HZSM-5 support; its shape selectivity favors the overcracking of liquid-range products to smaller gaseous products.^44^

From the second hour onward, the PtPd/HZSM-5 catalyst showed a significant decline in activity, reducing the overcracking of products. This leads to an increase in the selectivity to gasoline of 44.1 wt%. After the initial deactivation, the catalyst reached a constant selectivity of LPG. This stability can be attributed to the β-scission reactions occurring within the catalyst, which are promoted by the presence of strong acid sites of the HZSM-5 framework.^34^ On the other hand, the PtPd/HY catalyst displayed a more gradual activity loss. As the production of LPG decreased, the selectivity of gasoline increased up to 81.7 wt%. Notably, the gasoline selectivity from this catalyst almost doubled that achieved with the PtPd/HZSM-5 catalyst. The larger cavities present in the PtPd/HY zeolite facilitated a greater selectivity of gasoline-range hydrocarbons (C_5_–C_12_).^45^ The faster initial deactivation of the PtPd/HZSM-5 catalyst suggests that coke blocks the narrower pores of the HZSM-5 zeolite more easily (Table 2), which is also favored by the stronger acidity of the HZSM-5 (Figure 1b). This restricts the diffusion of heavier feed components to the active sites.

From a comparison of the two catalysts supported on the HY zeolite, the NiW/HY catalyst exhibited only slightly lower conversion at the beginning of the reaction (Figure 2c) but showed markedly lower activity for overcracking to LPG and dry gas. Consequently, the pseudo-steady-state is stabilized earlier than the PtPd/HY catalyst, with a steady conversion of ca. 60 wt% and a gasoline selectivity of 69 wt%, only 12 wt% lower than that of the PtPd/HY catalyst at TOS 8 h. When the behavior of both catalysts is compared, it becomes evident that the NiW/HY catalyst experienced a more pronounced activity decay than the PtPd/HY catalyst. Considering that transition metal catalysts generally display higher tolerance to sulfur-containing feeds,^46^ we confirmed that coking is the main source of deactivation. Due to the poorer porous texture of the NiW/HY, caused by the higher amount of deposited metals (Table 2), it deactivates faster. Moreover, the NiW function is less active than the PtPd function for the hydrogenation of coke precursors. Nevertheless, both HY-based catalysts showed gasoline selectivity higher than that achieved with the PtPd/HZSM-5.

The composition of the liquid products was characterized using PIONA analysis, which identified n-paraffins, i-paraffins, olefins, naphthenes, and aromatics. To further explain these results, it is important to understand the hydrocracking mechanism for each family of compounds.^47^ The aromatics present in the feed are initially hydrogenated at the metallic sites to form their corresponding naphthenes. These naphthenes then diffuse to the acid sites, where ring-opening reactions occur to produce olefins. Subsequently, these olefins are hydrogenated on the metallic sites, mostly generating n-paraffins. Inversely, n-paraffins may dehydrogenate on these sites to olefins, which is less likely due to the high partial pressure of H_2_. Olefins can also be protonated on the Brønsted acid sites to form carbenium ions, which are easily isomerized and hydrogenated to produce i-paraffins.

The feedstock is mainly formed by diaromatics and monoaromatics (Figure 3d–f). Due to the size of the zeolite pores, monoaromatics may be able to diffuse in and react, while diaromatics would need to react on the external surface of the catalyst. As observed, the PtPd/HZSM-5 barely decreased the amount of diaromatics, suggesting a negligible hydrocracking of the feedstock. In fact, after 2 h on stream, the concentration of both mono and diaromatics was higher than in the feed, suggesting a significant blockage of the zeolite pores and high activity for aromatization reactions.^48^ This substantially changes when the PtPd/HY catalyst is used (Figure 3e). Here, diaromatics conversion is very high and the increase in monoaromatics goes together with an increase in i-paraffins, which suggests a higher activity for the cracking mechanisms. A combination of the wider porosity (Table 2) and weaker activity (Figure 1b) explains this increase in the hydrocracking activity that ultimately leads to a decrease in the total aromatic content.^24,49^ i-Paraffins reached concentrations of 30.1 wt% for PtPd/HY catalyst and 24.7 wt% for NiW/HY catalyst.

The effects of the metal function were not as significant as those of the zeolite. A similar composition of the products is observed during the initial hours of streamflow for the PtPd/HY and the NiW/HY catalysts. The faster deactivation of the NiW/HY led to a faster increase in diaromatics (main compounds in the feedstock) and a decrease in i-paraffins and monoaromatics. The decline in hydrogenation capability of the NiW/HY catalyst became evident from the second hour onward. When comparing the two HY-supported catalysts, the PtPd/HY catalyst yielded the lowest aromatic content, making evident the highest activity for hydrogenation of the noble metal catalysts. The combination of the moderate acid strength of the HY zeolite with the PtPd metallic phase resulted in a good combination for aromatic hydrogenation and showed slower deactivation.

The results indicate that the PtPd/HY catalyst is the most suitable for hydrocracking of the B20 blend. The combination of noble metals with HY zeolite maximized gasoline production while minimizing the aromatic content. Additionally, sulfur poisoning of the noble metals did not appear to be a significant factor contributing to the loss of activity. The incorporation of the PPO may have aided in reducing the sulfur concentration, which was not a critical issue for catalyst deactivation. Instead, blockage of the porous structure seemed to be a more crucial factor.

#### 3.2.2 Effect of Pressure

After establishing that the PtPd/HY catalyst offered the best performance in the hydrocracking of the B20 blend, the impact of increasing the operating pressure was explored. All the experimental variables were maintained constant at 400 °C, an H_2_:feed ratio of 2000:1, a space-time of 0.23 g h g_feed_^–1^, and a reaction time of 8 h, with the only variable adjusted being pressure, which was increased to 80 bar.

The increase in pressure did not substantially influence the conversion of the heavy fraction of the feed; consequently, the selectivity of diesel and gasoil remained stable. This finding is consistent with the observations of Mosio-Mosiewski and Morawski,^50^ who reported similar results when processing heavy and highly aromatic feeds. Nevertheless, the elevated pressure had a more pronounced effect on the formation of lighter products. At the beginning of the reaction, gas selectivity was slightly lower at 80 bar (Figure S1) compared to 60 bar (Figure 3b), as cracking reactions are favored at low pressure. Thus, an increased production of gasoline was obtained at 80 bar. Nonetheless, the increase in total pressure and the unfavored cracking reaction resulted in slightly lower conversion values (Figure S2). Despite this trend, a subtle variation was obtained, with gasoline selectivity being 23 wt% at 60 bar and 30 wt% at 80 bar at TOS 1 h. The influence of pressure became more evident at 8 h of TOS, where a variation in the catalyst stability is also observed. Figure 4 shows the increase in the formation of each lump, which is defined as the difference between the concentration of each lump in the products and in the feed at the end of the reaction. Notably, an increase in LPG selectivity of 8 wt% was observed at 80 bar compared to negligible gas formation at 60 bar (Figure 4a,b respectively). Conversely, the maximum gasoline selectivity was achieved at the lower pressure of 60 bar, increasing its selectivity by 56 wt% at 60 bar and 46 wt% at 80 bar. Moreover, the increase in the partial pressure of H_2_ enhanced the residual activity of the catalyst,^51^ resulting in overcracking of the gasoline fraction at 80 bar.

**Figure 4:**
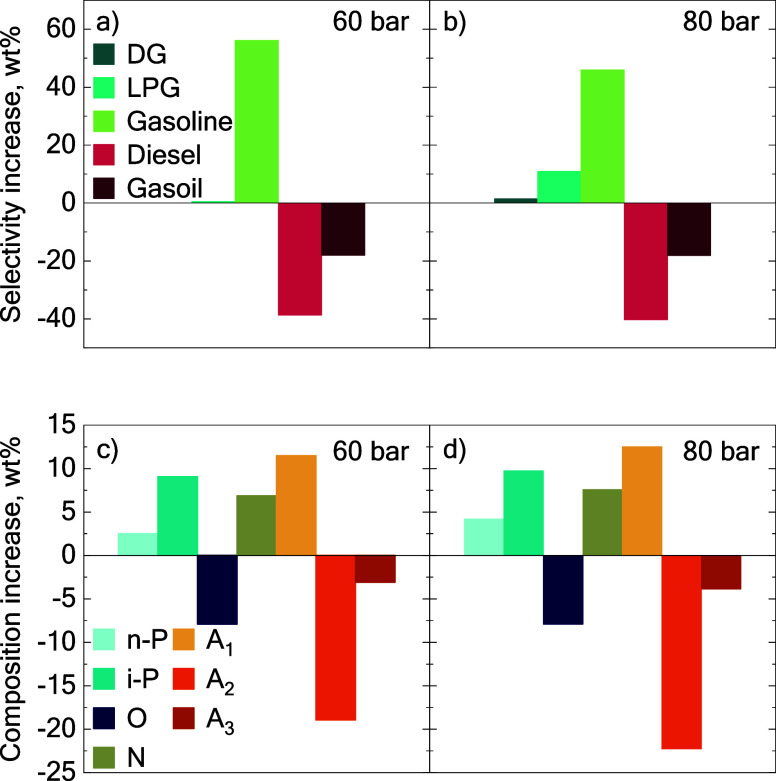
Effect of pressure on product selectivity increase (a, b) and composition increase (c, d) at TOS 8 h compared to the fractions of the feed. The hydrocracking reactions were performed at 400 °C with a PtPd/HY catalyst using the B20 blend as feedstock. Key: n-P, n-paraffins; i-P, i-paraffins; O, olefins; N, naphthenes; A_1_, monoaromatics; A_2_, diaromatics; A_3_, polyaromatics.

Regarding the product composition at the end of the reaction (Figure 4c,d), the observed differences were relatively small. The increase in pressure slightly improved the removal of diaromatics (a decrease of less than 4 wt% in concentration), which was reflected in the increments of n-paraffins (2 wt%), i-paraffins (1 wt%), and monoaromatics (1 wt%).

A comparative analysis of the reactions conducted at 60 and 80 bar indicated that the pressure increase did not yield significant differences after reaction for 8 h of reaction. The enhanced residual activity of the catalyst at higher pressures led to a reduction in gasoline selectivity due to overcracking. Furthermore, there was no marked effect on the conversion of heavier compounds, resulting in similar conversions and product compositions under both operating conditions. Consequently, and also considering the increase in costs related to compression to reach 80 bar, it can be concluded that the most favorable pressure for the hydrocracking of the B20 blend is 60 bar.

#### 3.2.3 PPO Blending Ratios

The subsequent phase of the study involved evaluating the impact of the PPO blending ratio on the product distribution. To achieve this, we compared the B20 blend with a more concentrated blend in PPO, B40 (40 wt% PPO and 60 wt% LCO). For the sake of comparison, experiments were also performed with neat LCO, which, for consistency, was named B0. The results are depicted in Figure 5. The incorporation of PPO with LCO significantly altered the product distribution. Notably, an increase in the proportion of PPO in the feedstock was correlated with a greater formation of light compounds. Comparing the results from B0 (Figure 5a) to those from the B20 blend (Figure 5b), gasoline formation rose from 73.9 to 81.7 wt%. Conversely, while the B40 blend resulted in a decrease in gasoline selectivity (Figure 5c), there was a notable increase in LPG production (23.6 wt%). This result is attributed to the paraffinic and olefinic nature of the PPO (Table 1, 79.6 wt% combined). The cracking of these hydrocarbons into lighter compounds is easier and faster than the cracking of the components of the LCO.^52^ The dilution of the heavier fractions present in the feedstock leads to higher conversions (Figure 6). However, with the increase in PPO content up to 40 wt% (as in the B40 blend), there was an increase in diesel selectivity, which rose by 6.7 wt% compared to the selectivity obtained from the B20 blend (23.6 vs 16.9 wt%). The distinct chemical properties of LCO and PPO play a crucial role in this process, as olefins derived from the PPO compete with aromatics from the LCO for preferential adsorption on acid sites.^53^ Given the high reactivity of olefins during hydrocracking, the conversion of PPO was favored over LCO, increasing the gas selectivity,^54,55^ and hindering the combined conversion of LCO and PPO. As a result, the highest overall hydrocracking conversion was observed with the B20 blend.

**Figure 5:**
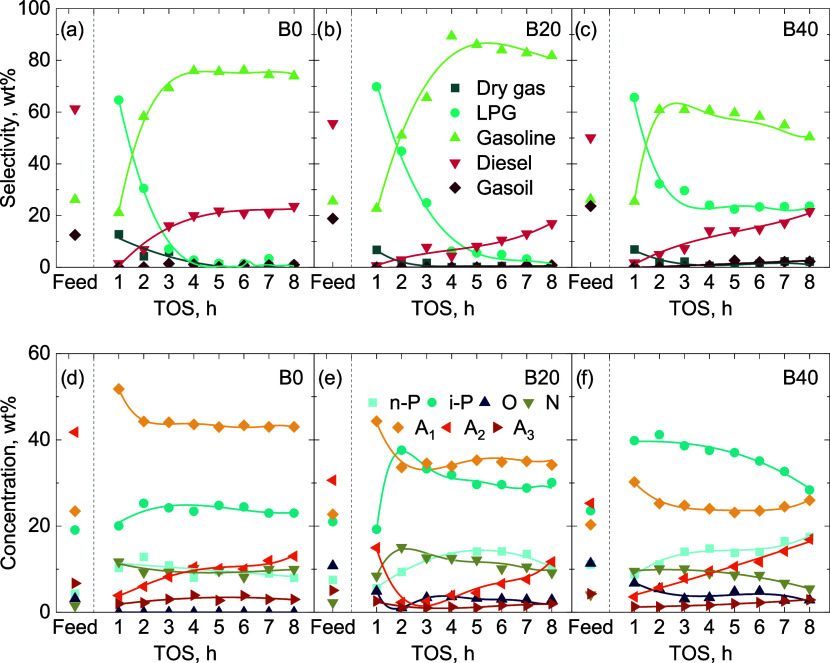
Effect of the PPO blending ratio on the evolution with the TOS of the product selectivity (a–c) and the composition (d–f). The hydrocracking reactions were performed at 400 °C and 60 bar using the PtPd/HY catalyst. Key: n-P, n-paraffins; i-P, i-paraffins; O, olefins; N, naphthenes; A_1_, monoaromatics; A_2_, diaromatics; A_3_, polyaromatics.

**Figure 6:**
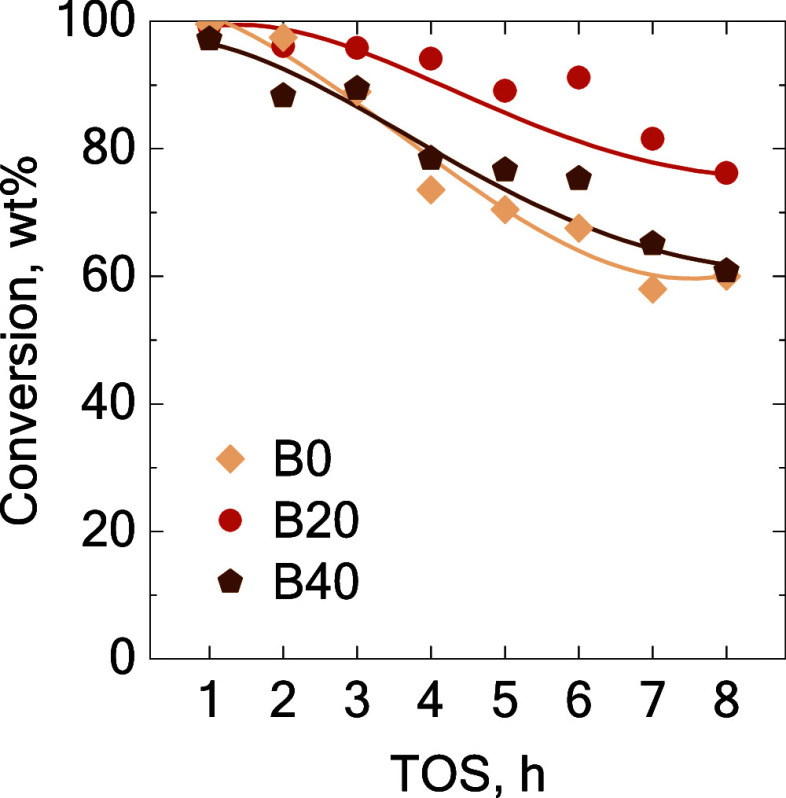
Evolution of the conversion with the TOS. Comparison between feeds with PtPd/HY catalyst at 400 °C and 60 bar.

In line with the previous observations, the analysis of product composition (Figure 5d–f) indicates that a higher proportion of PPO in the feedstock favored the formation of paraffins, mainly i-paraffins. An assessment of their evolution with reaction time revealed a notable decrease in the concentration of i-paraffins alongside an increase in n-paraffins. Concurrently, the concentration of monoaromatics decreased, whereas diaromatics exhibited an upward trend, surpassing the levels obtained with the other blends. Interestingly, the concentration of diaromatics after 8 h on stream is much closer to the feedstock concentration than that obtained with the B20 blend. This suggests hindered hydrogenation of these compounds due to the presence of olefins from the PPO, which leads to lower conversion of this blend (Figure 6).

The results suggest that the cofeeding of PPO with LCO has the potential to enhance fraction distribution; however, its extent is highly influenced by operating conditions and blending ratios used. Notably, the high concentration of PPO in the B40 blend promoted cracking reactions, which significantly increased the gas production. On the contrary, the blockage of the catalyst acid sites reduced conversion rates and gasoline selectivity. Therefore, the proportion of PPO in the B20 blend is deemed to be more suitable for optimizing both conversion and gasoline production.

### 3.3 Catalyst Deactivation by Coke

In the previous sections, a loss of catalyst activity during the reaction was observed, which was mainly assigned to the formation of coke. TPO analyses of the spent catalysts were conducted to gather information on the extent of the deactivation due to coke and the potential regeneration of the catalysts. The differential thermogravimetric (DTG) curves are presented in Figure 7. These curves, along with the peak maxima, provide valuable information about the nature and location of the deposited coke.

**Figure 7:**
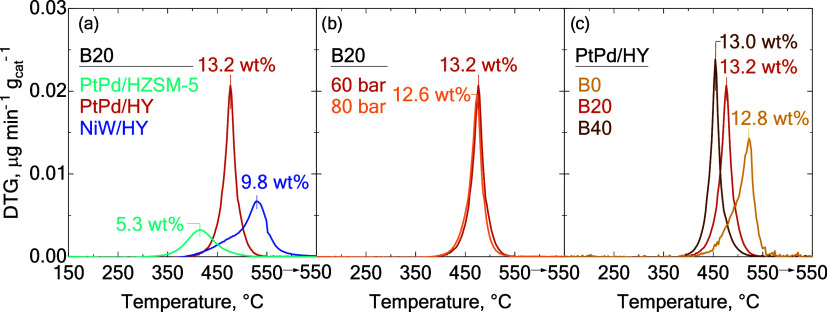
TPO profiles of the coke formed. (a) Comparison between catalysts using B20 blend at 400 °C and 60 bar, (b) comparison between working pressure with PtPd/HY catalyst at 400 °C, and (c) comparison between feeds with PtPd/HY catalyst at 400 °C and 60 bar. The total amount of coke is displayed according to the color of the legend.

The TPO profiles of the three catalysts employed in the hydrocracking of the B20 blend are shown in Figure 7a. The total coke content ranged from 5.0 to 13.2 wt%, with the temperature peaks varying from 419 to 532 °C, indicating notable differences among the catalysts. The PtPd/HZSM-5 catalyst exhibited the lowest coke deposition (5.3 wt%), which might be attributed to the smaller pore size of the zeolite,^56^ which limits the formation of bulky reaction intermediates.^34^ The peak temperature and the small amount of coke suggest that this coke may be external, as a consequence of the condensation of big molecules of the feed that cannot diffuse into the zeolite micropores. This hypothesis agrees with the very low conversion of diaromatics with this catalyst (Figure 3a). Conversely, the PtPd/HY catalyst presented the highest coke deposition (13.2 wt%). The greater coke content observed with HY zeolite in comparison to HZSM-5 zeolite could be attributed to the structural characteristics and larger pore sizes of HY zeolite. But most likely it agrees with the condensation of species inside the pores of the zeolite as a result of the cracking mechanism. This leads to the accumulation of coke within the zeolite pores, which is consistent with the observed peak at 477 °C.

Comparing the coke formation on both HY zeolite-supported catalysts, it was observed that the NiW/HY catalyst exhibited a lower amount of deposited coke (9.8 wt%) than the PtPd/HY catalyst (13.2 wt%). This result can be attributed to the relatively lower acid strength of the NiW/HY catalyst. Indeed, previous studies have demonstrated that coke formation is significantly influenced by the strength of the acid sites present on the catalyst.^39^ Distinctively, the TPO profile of the NiW/HY catalyst was the only one that could be deconvoluted into Gaussian-type contributions. The primary contribution, located at 480 °C, indicated that the nature and location of the coke were analogous to those observed with the PtPd/HY catalyst. Contrarily, the contribution observed at a higher temperature of 532 °C suggested the presence of more developed carbonaceous structures. The appearance of this second peak could be associated with the lower hydrogenation activity of transition metals, which may have led the coke to condense, resulting in low H/C ratio structures.^57^

Increasing the pressure to 80 bar slightly reduced coke deposition (Figure 7b) when using the PtPd/HY catalyst, decreasing it from 13.2 wt% at 60 bar to 12.6 wt% at 80 bar. The higher hydrogen pressure in the reaction medium contributed to the hydrocracking of coke precursors and heavy compounds,^58^ in line with the previously discussed liquid phase results (Figures 4b and S2).

The DTG curves of the used PtPd/HY catalyst with blends B0, B20, and B40 are depicted in Figure 7c. The TPO profiles showed different shapes with the incorporation of the PPO into the feedstock. In line with the composition of the liquid products, an increase in the PPO blending ratio in the feed reduced the aromatic content in the reaction medium. Consequently, the deposited coke is less developed, which is characterized by a decrease in the temperatures of the DTG peaks.^59^ Interestingly, although the overall amount of coke deposited with the B0 blend (12.6 wt%) was lower than that from the B20 (13.2 wt%) and B40 (13.0 wt%) blends, the nature of the more developed coke may have a greater influence on catalyst deactivation than the quantity of deposited coke. Moreover, the use of the blend decreases the needed temperature and time for the regeneration of the catalyst.

### 3.4 Catalyst Regenerability

The reusability of the catalyst was studied by using both LCO and the B20 blend. The PtPd/HY catalyst was selected for this study, as it exhibited the highest activity in terms of maximizing the gasoline yield while simultaneously reducing the aromatic content of the products. The procedure entailed conducting the reaction with the fresh catalyst for 6 h, followed by catalyst regeneration and repetition under identical pressure and temperature conditions. In the second cycle, however, the reaction time was shortened to 3 h, which was sufficient to detect potential deviations in catalytic behavior. Catalyst regeneration was carried out by calcination in air at 450 °C (calcination temperature) for 6 h with a flow rate of 30 cm^3^ min^–1^. This protocol was shown to be effective in removing most of the coke in the thermobalance (Figure S3).

Figure 8 depicts the evolution of product selectivity and hydrodearomatization conversion (*X*_HDA_) as a function of the time on stream. Regarding the LCO feed (Figure 8a), the selectivity pattern obtained after regeneration indicates partial recovery of catalytic activity, as the regenerated catalyst did not follow the trend observed at 6 h of TOS with the fresh catalyst. Nevertheless, the efficiency of the hydrocracking function diminished relative to the initial state. Gas and gasoline selectivities decreased, whereas the diesel fraction increased. Notably, the diesel selectivity at 3 h TOS with the regenerated catalyst (20.9 wt%) matched that of the fresh catalyst after 6 h of reaction. A similar behavior was observed when processing the B20 blend (Figure 8c). Even though the regenerated catalyst remained active, a significant decline in cracking reactions was detected, accompanied by an increase in heavy fractions.

**Figure 8:**
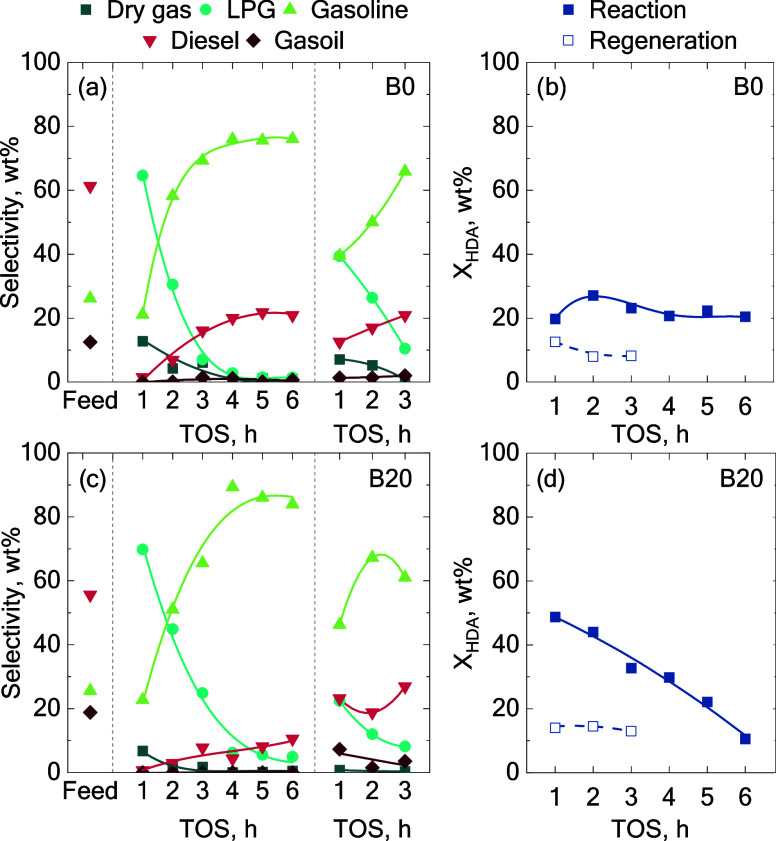
Effect of PtPd/HY catalyst regeneration (a, c) in the product selectivity and (b, d) HDA conversions with LCO and B20 blend.

HDA conversion was defined from the total amount of aromatics in the products and feed to assess the activity of the hydrogenation function in the different cycles. As shown in Figure 8b, the activity exhibited during the first reaction cycle was not restored after regeneration. This fact could indicate that the metal function underwent irreversible deactivation, caused by sintering of the metal particles, sulfur poisoning, or metal deposition.^60^ In contrast, the HDA conversion obtained with the B20 blend (Figure 8d) surpassed the value obtained at 6 h TOS with the fresh catalyst (10.6 wt%), reaching 14.0 wt%. Although the recovered activity did not attain the initial level, a partial restoration of the hydrogenation function was observed, likely attributable to the dilution of the potentially poisonous species (sulfur and metals) in the B20 feed. Further characterization of catalysts, potentially with higher amounts of PtPd to achieve results above the error threshold, would be needed to exactly disclose the reason for the partial irreversible deactivation. This is beyond the scope of this work.

## 4 Conclusions

To promote the recycling of plastics at an industrial scale, this study investigated the hydrocracking of HDPE-derived PPO blended with LCO, a byproduct from refineries, using a fixed-bed reactor. Various catalysts were studied to assess the influence of their properties, including acidity and structural characteristics. The PtPd/HZSM-5 and PtPd/HY catalysts demonstrated similar density of acid sites; however, the higher strength of the HZSM-5 zeolite was associated with a higher propensity for overcracking, yielding a gas output of 10 wt% during the pseudo-steady-state operation. Moreover, the shape selectivity of the HZSM-5 zeolite impeded the diffusion of bulkier molecules from the feedstock to the active sites, resulting in smaller conversion extents and faster deactivation by coke.

In contrast, the PtPd/HY catalyst, characterized by a high micropore volume and a structural arrangement composed of larger cavities, eased the production of a greater amount of gasoline-range hydrocarbons, achieving a best selectivity of 81 wt% at steady conditions. The NiW/HY catalyst also exhibited good performance, providing a gasoline selectivity of 69 wt% while demonstrating a lower deactivation rate than its noble metal analogues. However, its lower hydrogenation activity contributed to an elevated aromatic content.

Further experimentation with the PtPd/HY catalyst at an increased pressure of 80 bar led to excessive gas production without enhancing the conversion rates. A comparative analysis of different blending ratios indicated that when the proportion of PPO in the blend reached 40 wt% (B40 along the text), a similar reaction behavior was observed. The feed compounds of PPO, mainly paraffins and olefins, showed a higher reactivity compared to those of LCO, easing their cracking into lighter products. Consequently, gas selectivity increased at the same time that gasoline production and conversion decreased. A blend comprising 20 wt% of PPO (designated as B20) showed a good balance between the two feeds, maximizing conversion and gasoline selectivity and favoring hydrodearomatization reactions, thus enhancing the content of i-paraffins relative to the neat LCO feed. Furthermore, the incorporation of PPO resulted in the formation of less condensed coke, attributable to the lower aromatic concentration observed in the product stream.

In conclusion, the best results in terms of selectivity and gasoline quality were achieved through hydrocracking the B20 blend (20 wt% of PPO and 80 wt % of LCO) using the PtPd/HY catalyst under a pressure of 60 bar. These findings underscore the viability of blending plastics pyrolysis oils with refinery streams as a sustainable strategy for plastic waste recovery at an industrial level.

## Supplementary Material



## Notes

Properties of the PPO/LCO blends; evolution with the TOS of the product selectivity and the composition obtained in the hydrocracking of the B20 blend; evolution of the conversion with the TOS; comparison between working pressures; regeneration test in the thermobalance of the used PtPd/HY catalyst (PDF)

## §

Department of Chemical Engineering, Andalucía Tech, University of Malaga, Campus de Teatinos s/n, 29010 Málaga, Spain

## References

[1] Abbas S. N., Qureshi M. I. (2025). Effect of recycled
plastic aggregates on mechanical
and durability properties of concrete: A review. Mater. Chem. Phys.: Sustain. Energy.

[2] Plastics Europe . Plastics Europe. Plast Facts 2024, 2024; 1.

[3] Agrawal M., Vianello A., Picker M., Simon-Sánchez L., Chen R., Estevinho M. M. (2024). Micro- and nano-plastics,
intestinal inflammation, and inflammatory bowel disease: A review
of the literature. Sci. Total Environ..

[4] Cusba J., Pacheco C., Espinosa-Díaz L., Laude C., Fuerst L., Obando-Madera P. (2026). Plastic pollution in
marine coastal areas: Quantifying leakage and evaluating management
responses. Mar. Pollut. Bull..

[5] Huang J., Veksha A., Lisak G., Nie B. (2025). Consecutive coproduction
of H2 and syngas from waste polyolefins derived pyrolysis gas via
chemical looping cracking–gasification. Waste Manage..

[6] Zhang J., Chu Z., Cao R., Wu X., Han K. (2026). A review of resource
recovery from waste plastics via pyrolysis and gasification. Fuel.

[7] Peng Y., Wang Y., Ke L., Dai L., Wu Q., Cobb K. (2022). A review
on catalytic pyrolysis of plastic wastes to
high-value products. Energy Convers. Manage..

[8] Lee J., Kwon E. E., Lam S. S., Chen W.-H., Rinklebe J., Park Y.-K. (2021). Chemical recycling
of plastic waste via thermocatalytic
routes. J. Cleaner Prod..

[9] Qureshi M. S., Oasmaa A., Pihkola H., Deviatkin I., Tenhunen A., Mannila J. (2020). Pyrolysis
of plastic
waste: Opportunities and challenges. J. Anal.
Appl. Pyrolysis.

[10] Yoon B. S., Kim C., Park G.-J., Jeon S. G., Ko C. H. (2024). Upgrading waste
plastic pyrolysis oil via hydrotreating over sulfur-treated Ni-Mo/Al_2_O_3_ catalysts. Fuel.

[11] Palos R., Crespo I., Trueba D., Klimov O. V., Kazakov M. O., Bilbao J., Gutiérrez A. (2024). Hydrocracking
of plastic pyrolysis
oil (PPO) blended with vacuum gas oil (VGO) over Pt–Pd catalysts
supported on USY–ASA–Al_2_O_3_ composites. Energy Fuels.

[12] Lim S. H., Pham H. H., Kwon E. H., Nho N. S. (2025). Optimizing the use
of pyrolysis waste oil as a feedstock for the naphtha cracking process
by hydrotreating and hydrocracking. Resour.,
Environ. Sustainability.

[13] Palos R., Gutiérrez A., Vela F. J., Olazar M., Arandes J. M., Bilbao J. (2021). Waste Refinery: The Valorization
of Waste Plastics
and End-of-Life Tires in Refinery Units. A Review. Energy Fuels.

[14] Speight, J. G. Hydrotreating and Hydrocracking Processes in Refining Technology; CRC Press: Boca Raton, 2023.

[15] Trueba D., Palos R., Bilbao J., Arandes J. M., Gutiérrez A. (2023). Kinetic modeling
of the hydrocracking of polystyrene blended with vacuum gasoil. Chem. Eng. J..

[16] Hao Y., Che X., Hou X., Shi L., Huang L. (2025). Recent Advances
in
the Thermo-Catalytic Upcycling of Polyethylene Waste. ACS Appl. Polym. Mater..

[17] Goh G. K. H., Dai J. E. C. M. (2025). A Mini-review on Development for the Direct Catalytic
Hydroconversion of Polypropylene to Liquid and Gaseous Hydrocarbons
for Fuels and Chemicals. Top. Catal..

[18] Kim C., Yoon B. S., Park G.-J., Kwon E. H., Pham H. H., Lim S. H. (2025). Catalytic
hydrocracking of waste plastic pyrolysis
oil for production of high-quality naphtha over NiMo/mesoporous HZSM-5
catalyst. Fuel.

[19] Kim K. D., Kwon E. H., Kim K. H., Lim S. H., Pham H. H., Go K. S. (2023). Study
of Hydrotreating and Hydrocracking Catalysts
for Conversion of Waste Plastic Pyrolysis Oil to Naphtha. Appl. Chem. Eng..

[20] Rodríguez S., Trueba D., Escribano M., Arandes J. M., Palos R., Gutiérrez A. (2025). Six-lump kinetic
model for plastic pyrolysis oil (PPO)
and vacuum gasoil (VGO) blend hydroprocessing considering selective
catalyst deactivation. Catal. Today.

[21] Thangaraj B., Lee Y. (2025). Recent advancements
in upcycling of polyolefins by hydrocracking:
Effect of various heterogeneous catalysts and reaction parameters. Polym. Degrad. Stab..

[22] Aydogdu A. C., Erkmen B., Suerkan A., Ezdesir A., Guliyev B., Celik G. (2024). Chemical upcycling
of polyolefins into liquid refinery feedstock
from the circularity and chemical engineering aspects. J. Environ. Chem. Eng..

[23] Fahim, M. A. ; Alsahhaf, T. A. ; Elkilani, A. Hydroconversion. In Fundamentals of Petroleum Refining; Elsevier, 2010; pp 153–198.

[24] Alabi-Babalola O., Asuquo E., Alhassawi H., Alnasser M., Nadri A., Chansai S. (2026). Selective hydrocracking of polystyrene waste over Pt-
and Ru-supported zeolite catalysts into high yield LPG and ethylbenzene. Fuel Process. Technol..

[25] Liu J., Zhong X., Gao L., Zhang Y., Wang Z., Shakeri M. (2025). Hydrocracking of polyethylene to high quality liquid
fuels over bimetallic catalyst PdAg/HZSM-5. Microporous Mesoporous Mater..

[26] Dai X., Cheng Y., Liu T., Mao L. (2024). Catalyst for moderate
hydrocracking of polycyclic aromatic hydrocarbons in LCO to produce
chemical raw materials: Ni in-situ modified Y zeolite-supported-NiWS. J. Ind. Eng. Chem..

[27] Rodríguez S., Palos R., Crespo I., Bilbao J., Arandes J. M., Gutiérrez A. (2025). Suitable properties of the HY zeolite
of NiW/HY catalysts
for the hydroprocessing of a plastic pyrolysis oil/vacuum gas oil
(PPO/VGO) blend. J. Anal. Appl. Pyrolysis.

[28] Crespo I., Palos R., Trueba D., Bilbao J., Arandes J. M., Gutiérrez A. (2023). Intensifying
gasoline production in the hydrocracking
of pre-hydrotreated light cycle oil by means of Pt and Pd supported
on a spent FCC catalyst. Fuel.

[29] Batalha N., Comparot J.-D., Le Valant A., Pinard L. (2022). In situ FTIR spectroscopy
to unravel the bifunctional nature of aromatics hydrogenation synergy
on zeolite/metal catalysts. Catal. Sci. Technol..

[30] Palos R., Gutiérrez A., Vela F. J., Maña J. A., Hita I., Asueta A. (2019). Assessing the potential
of the recycled plastic slow pyrolysis for the production of streams
attractive for refineries. J. Anal. Appl. Pyrolysis.

[31] Palos R., Gutiérrez A., Arandes J. M., Bilbao J. (2018). Catalyst used in fluid
catalytic cracking (FCC) unit as a support of NiMoP catalyst for light
cycle oil hydroprocessing. Fuel.

[32] van
Herk D., Castaño P., Quaglia M., Kreutzer M. T., Makkee M., Moulijn J. A. (2009). Avoiding segregation during the loading
of a catalyst–inert powder mixture in a packed micro-bed. Appl. Catal., A.

[33] Valle B., Palos R., Bilbao J., Gayubo A. G. (2022). Role of
zeolite
properties in bio-oil deoxygenation and hydrocarbons production by
catalytic cracking. Fuel Process. Technol..

[34] Costa C. S., Thi H. D., Van Geem K. M., Ribeiro M. R., Silva J. M. (2022). Assessment
of acidity and the zeolite porous structure on hydrocracking of HDPE. Sustainable Energy Fuels.

[35] Riaz S., Ahmad N., Farooq W., Ali I., Sajid M., Akhtar M. N. (2025). Catalytic pyrolysis of HDPE for enhanced
hydrocarbon
yield: A boosted regression tree assisted kinetics study for effective
recycling of waste plastic. Digital Chem. Eng..

[36] Han Z., Zhou F., Zhao J., Liu Y., Ma H., Wu G. (2020). Synthesis of hierarchical GaZSM-5
zeolites by a post-treatment method
and their catalytic conversion of methanol to olefins. Microporous Mesoporous Mater..

[37] Kostyniuk A., Bajec D., Prašnikar A., Likozar B. (2021). Catalytic hydrocracking,
hydrogenation, and isomerization reactions of model biomass tar over
(W/Ni)-zeolites. J. Ind. Eng. Chem..

[38] Hu Z., Wu T., Xie H., Zhang Y., Ge S., Wu Z. (2024). Synthesis
and consequence of three-dimensionally ordered macroporous Beta zeolite
supported NiW catalyst for efficient hydrocracking of 1-methylnaphthalene
to BTX. Chem. Eng. J..

[39] Kostyniuk A., Bajec D., Likozar B. (2022). Catalytic
hydrocracking reactions
of tetralin biomass tar model compound to benzene, toluene and xylenes
(BTX) over metal-modified ZSM-5 in ambient pressure reactor. Renewable Energy.

[40] Vivas-Báez J. C., Servia A., Pirngruber G. D., Dubreuil A.-C., Pérez-Martínez D. J. (2021). Insights
in the phenomena involved in deactivation of industrial hydrocracking
catalysts through an accelerated deactivation protocol. Fuel.

[41] Furimsky E. (1999). Deactivation
of hydroprocessing catalysts. Catal. Today.

[42] Saab R., Damaskinos C. M., Polychronopoulou K., Efstathiou A. M., Charisiou N., Goula M. (2022). Ni/CNT/Zeolite-Y composite
catalyst for efficient heptane hydrocracking: Steady-state and transient
kinetic studies. Appl. Catal., A.

[43] Vela F. J., Palos R., Trueba D., Bilbao J., Arandes J. M., Gutiérrez A. (2021). Different
approaches to convert waste polyolefins into
automotive fuels via hydrocracking with a NiW/HY catalyst. Fuel Process. Technol..

[44] Azam M. U., Fernandes A., Ferreira M. J., McCue A. J., Graça I., Afzal W. (2025). Unlocking the structure-activity
relationship of hierarchical MFI
zeolites towards the hydrocracking of HDPE. Fuel.

[45] Costa C. S., Muñoz M., Ribeiro M. R., Silva J. M. (2021). H-USY and H-ZSM-5
zeolites as catalysts for HDPE conversion under a hydrogen reductive
atmosphere. Sustainable Energy Fuels.

[46] Anilkumar M., Loke N., Patil V., Panday R., G S. (2020). Hydrocracking
of hydrotreated light cycle oil to mono aromatics over non-noble bi-functional
(Ni-W supported) zeolite catalysts. Catal. Today.

[47] Weitkamp J. (2012). Catalytic
Hydrocracking-Mechanisms and Versatility of the Process. ChemCatChem.

[48] Qie Z., Xiang H., Xiang H., Zou R., Alhelali A., Alhassawi H. (2024). Catalytic pyrolysis of high-density polyethylene
(HDPE) over hierarchical ZSM-5 zeolites produced by microwave-assisted
chelation-alkaline treatment. Fuel.

[49] Oh Y., Noh H., Park H., Han H., Nguyen T. B., Lee J. K. (2020). Molecular-size
selective hydroconversion of FCC light cycle oil into petrochemical
light aromatic hydrocarbons. Catal. Today.

[50] Mosio-Mosiewski J., Morawski I. (2005). Study on single-stage
hydrocracking of vacuum residue
in the suspension of Ni–Mo catalyst. Appl. Catal., A.

[51] Gutiérrez A., Arandes J. M., Castaño P., Olazar M., Bilbao J. (2012). Effect of
pressure on the hydrocracking of light cycle oil with a Pt–Pd/HY
catalyst. Energy Fuels.

[52] Tanimu A., Aitani A., Al-Shuqaih R. H., Alghamdi A. A., Alhassan A. M., Shafi S. (2024). Tuning the morphology
and textural properties of ZSM-5 additive for
co-cracking of waste plastics with vacuum gas oil to light olefins. Waste Manage..

[53] Che Y., Shi K., Wang Q., Tang R., Tian Y. (2022). Molecular
coupling
behavior of relay catalytic upgrading of heavy oil fast pyrolysis
vapor to produce light olefins. J. Anal. Appl.
Pyrolysis.

[54] Pleyer O., Vrtiška D., Straka P., Vráblík A., Jencík J., Šimácek P. (2020). Hydrocracking of a
heavy vacuum gas oil with fischer-tropsch wax. Energies.

[55] Gacem A., Sambandam P., Santhosh S., Silambarasan T., Saravanan P., Bairavi B. (2025). Catalytic hydroprocessing
of mixed plastic waste using Ni-Ce/ZSM-5: Performance and emission
analysis of diesel blends across blending ratios. Energy Rep..

[56] Marcilla A., Beltrán M. I., Hernández F., Navarro R. (2004). HZSM5 and HUSY deactivation
during the catalytic pyrolysis of polyethylene. Appl. Catal., A.

[57] Wang Y., Yan N., Chen Z. (2024). Identification of coke
species on Fe/USY catalysts
used for recycling polyethylene into fuels. RSC Adv..

[58] Vela F. J., Palos R., Bilbao J., Arandes J. M., Gutiérrez A. (2022). Hydrogen pressure
as a key parameter to control the quality of the naphtha produced
in the hydrocracking of an HDPE/VGO blend. Catalysts.

[59] Trueba D., Palos R., Crespo I., Veloso A., Azkoiti M. J., Bilbao J., Gutiérrez A. (2025). Production
of plastic-derived fuel
by cohydrocracking of different polyethylene terephthalate (PET) with
vacuum gas oil (VGO). Energy Fuels.

[60] Kohli K., Prajapati R., Maity S. K., Sau M., Garg M. O. (2016). Deactivation
of hydrotreating catalyst by metals in resin and asphaltene parts
of heavy oil and residues. Fuel.

