# Supplementary material for: Hydrocracking of Waste Plastic Pyrolysis Oil and Light Cycle Oil (PPO/LCO) Blends in a Trickle-Bed Reactor: Catalyst Assessment and Operating-Condition Screening
Source: Energy Fuels. 2026 Mar 30;40(14):7296–307. doi: 10.1021/acs.energyfuels.6c00240 (PMC13072571; doi:10.1021/acs.energyfuels.6c00240)
Supplement: Supplementary file 1 [file ef6c00240_si_001.pdf]

Hydrocracking of Waste Plastic Pyrolysis Oil and Light Cycle Oil (PPO/LCO) Blends in a Trickle-Bed Reactor: Catalyst Assessment and Operating-Condition Screening

Iratxe Crespo [a], Tomás Cordero-Lanzac [a, b, 1], Ana Cimiano [a], Roberto Palos [a,*], Alazne Gutiérrez [a]

[a] *Department of Chemical Engineering, University of the Basque Country UPV/EHU, PO Box 644, 48080 Bilbao, Spain*

[b] *IKERBASQUE, Basque Foundation for Science, 48009 Bilbao, Spain*

(*) corresponding author: roberto.palos@ehu.eus

[1] *Present address: Departamento de Ingeniería Química, Andalucía Tech, University of Malaga, Campus de Teatinos s/n 29010, Málaga, Spain*

Supporting information

Table S1. Properties of the PPO/LCO blends.

	**B20**	**B40**
Density, g cm^{-3}	0.879	0.859
Simulated distillation		
IBP–FBP, °C	101–488	154–366
T_{50}–T_{95},°C	260–413	243–348
Gasoline, wt%	25.6	26.3
Diesel, wt%	55.6	50.1
Gasoil, wt%	18.8	23.6
Lumped composition, wt%		
Paraffins	28.5	34.6
Olefins	10.8	11.6
Naphthenes	2.3	4.1
Aromatics	58.4	49.9
monoaromatics	22.7	20.3
diaromatics	30.6	25.3
polyaromatics	5.1	4.3

*B20: Blend composed of 20 wt% of PPO and 80 wt% of LCO.
*B40: Blend composed of 40 wt% of PPO and 60 wt% of LCO.

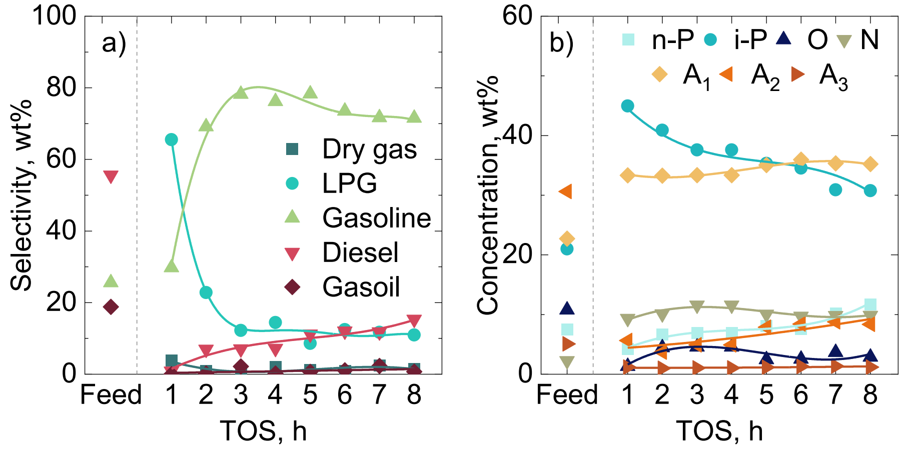

Fig. S1. Evolution with the TOS of (a) the product selectivity and (b) the composition obtained in the hydrocracking of the B20 blend at 400 °C and 80 bar with PtPd/HY catalysts. Key: n-P, n-paraffins; i-P, i-paraffins; O, olefins; N, naphthenes; A_1, monoaromatics; A_2, diaromatics; A_3, polyaromatics.

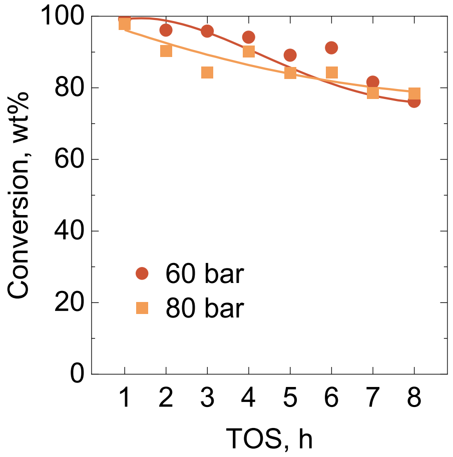

Fig. S2. Evolution of the conversion with the TOS. Comparison between working pressure with PtPd/HY catalyst at 400 °C.

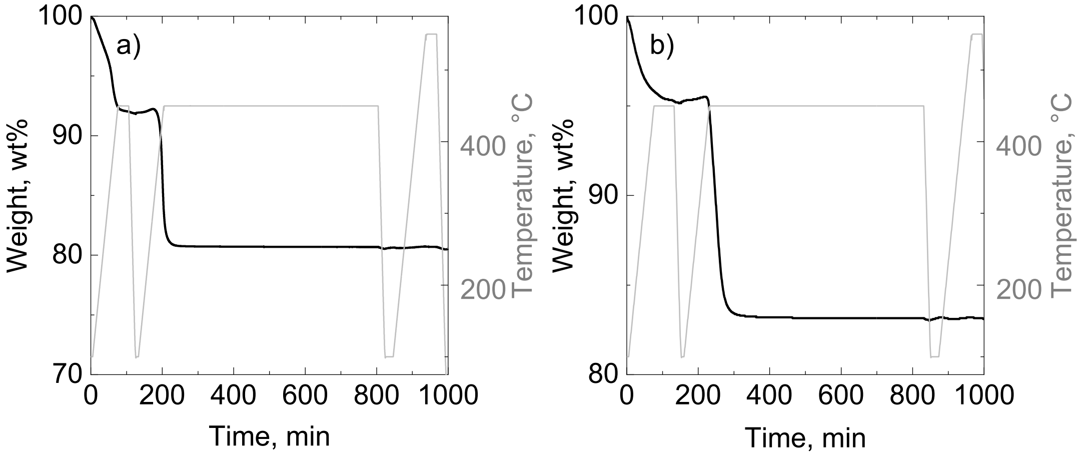

Fig. S3. Regeneration test in the thermobalance of the used PtPd/HY catalyst in the a) B0 and b) B20 hydrocracking.
